# Observational Study: Familial Relevance and Oncological Significance of Revised Bethesda Guidelines in Colorectal Patients That Have Undergone Curative Resection

**DOI:** 10.1097/MD.0000000000002723

**Published:** 2016-02-12

**Authors:** Won Beom Jung, Chan Wook Kim, Yong Sik Yoon, In Ja Park, Seok-Byung Lim, Chang Sik Yu, Jin Cheon Kim

**Affiliations:** From the Division of Colon and Rectal Surgery, Department of Surgery, University of Ulsan College of Medicine, and Asan Medical Center, Seoul, Republic of Korea.

## Abstract

Amsterdam criteria for the hereditary nonpolyposis colorectal cancer (HNPCC) exclude most suspect cases of possible hereditary colorectal cancer (CRC). By contrast, revised Bethesda guidelines excessively broaden the disease spectrum. The aim of this study is to retrospectively evaluate the cliniciopathilogical characteristics of patients fulfilling the revised Bethesda guidelines and to review the efficacy and limitations of the revised guidelines.

This retrospective study enrolled 3609 patients who underwent curative surgery for primary CRC. Patients were classified into the Bethesda group or the control group according to whether they fulfilled the revised Bethesda guidelines. Patients were further categorized when they fulfilled a minimum of 2 items of the revised guidelines. Individual items were analyzed for deficient mismatch repair (d-MMR).

The median follow-up was 82.9 (interquartile range, 72–101) months. Patients in the Bethesda group were younger and had a higher rate of reduced mismatch repair (MMR) protein expression, microsatellite instability, and right colonic involvement (all *P* < 0.001) than the control group. As a predictor of d-MMR, the revised Bethesda guidelines showed a sensitivity of 63.0% and a specificity of 72.6%. Items 1 and 2, respectively, or the item pair 1 and 2, were independent predictors of d-MMR (all *P* < 0.001). Patients fulfilling the Bethesda guidelines showed clinicopathological features of HNPCC.

The revised Bethesda guidelines appear to be a competent predictor of d-MMR. Specifically, items 1 and 2 are significant predictors of d-MMR and may be relevant to the application of the revised Bethesda guidelines.

## Abstract 2

Supplemental Digital Content is available in the text

## INTRODUCTION

Hereditary nonpolyposis colorectal cancer (HNPCC) is associated with a predisposition for colorectal, endometrial, gastric, urothelial, ovarian, pancreatic, sebaceous-gland, and other cancers.^1^ HNPCC is caused by germline mutations in mismatch repair (MMR) genes (*MLH1*, *MSH2*, *MSH6*, *MLH3*, *PMS2*, or *PMS1*), mainly *MSH2* and *MLH1*, although approximately 50% of individuals suspected of having HNPCC are not confirmed genetically.^2,3^ The prevalence of MMR germline mutations in the general population has been estimated at 1 in 300 to 500 individuals.^4^ Tumors arising in carriers of MMR gene mutations exhibit a characteristic phenotype termed microsatellite instability (MSI), which is characterized by alterations in the length of simple repetitive microsatellite sequences found throughout the genome.^5,6^ MSI is not specific for HNPCC-related tumors, as approximately 10% to 15% of sporadic colorectal cancer (CRC) exhibit MSI.^7^ When a somatic mutation inactivates the wild-type allele of the MMR gene, the tissue develops a hypermutable phenotype, which accelerates multi-step carcinogenesis.^8^ Tumor tissue obtained from HNPCC patients displays typical signs of deficient MMR (d-MMR), such as MSI with high frequency (MSI-H), along with reduced or lost expression of at least 1 MMR protein by immunohistochemical (IHC) staining.^9^

One of the strongest predictors of CRC is family history. A meta-analysis has shown a 2-fold higher risk in first-degree relatives of individuals diagnosed with CRC, and a 4-fold increased risk in relatives of individuals diagnosed before the age of 45 years.^10^ Furthermore, familial CRC can be comprehensively explained as multiple occurrences of colorectal and accompanying cancers, inherited via dominant or recessive transmission; family history may be correlated with MSI.^6,7^ In 1991, the Amsterdam criteria were originally designed to identify families appropriate for enrollment in research projects aimed at identifying the genetic causes of hereditary CRC.^11^ In 1999, these criteria were extended to extra-colonic cancers associated with HNPCC. However, even the revised Amsterdam II criteria have relatively low sensitivity (44–78%) for diagnosing HNPCC, although the Amsterdam criteria have high transmission of genetic penetrance. Therefore, in 1997, the National Cancer Institute hosted an international workshop to develop criteria to identify patients with CRC who should be offered MSI testing due to an increased risk for HNPCC and concurrently suggested the Bethesda guidelines with less stringent criteria.^12^ These guidelines considered the patient's medical and familial history of HNPCC-related tumors and early age of onset. In 2004, these guidelines were revised to achieve higher specificity (Table 1).^13^ Several retrospective studies reveal that 10.7% to 23.5% of patients with CRC fulfill at least 1 of the revised Bethesda guidelines.^2,14–17^ The Amsterdam criteria for HNPCC are strictly defined and exclude many cases of HNPCC suspects with hereditary trait. By contrast, the revised Bethesda guidelines excessively broaden the disease spectrum by including CRC families with specific accompanying cancers and clinicopathological characteristics. The revised Bethesda guideline is accordingly complex, making it difficult to take family history into account.

**TABLE 1:**
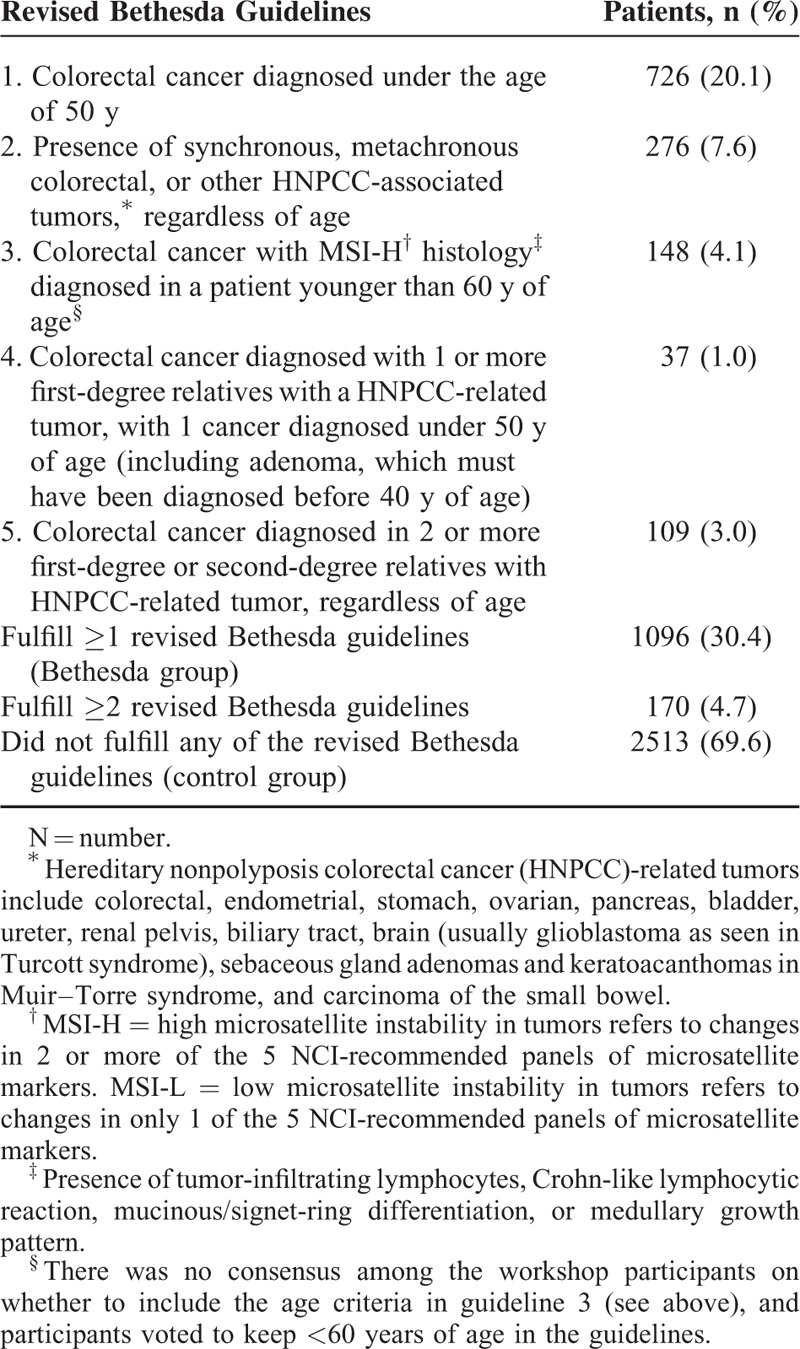
The Bethesda Groups in Patients Who Received Curative Resection for Colorectal Cancer^13,26^

The aim of this study was to retrospectively evaluate the clinicopathological characteristics of patients fulfilling the revised Bethesda guidelines and compare them with those of patients in a control group. We also reviewed efficacy and limitation of the revised Bethesda guidelines with respect to identify hereditary CRC. Furthermore, we aimed to reappraise individual items of revised Bethesda guidelines.

## MATERIALS AND METHODS

### Patient Enrollment and Exclusion Criteria

**FIGURE 1:**
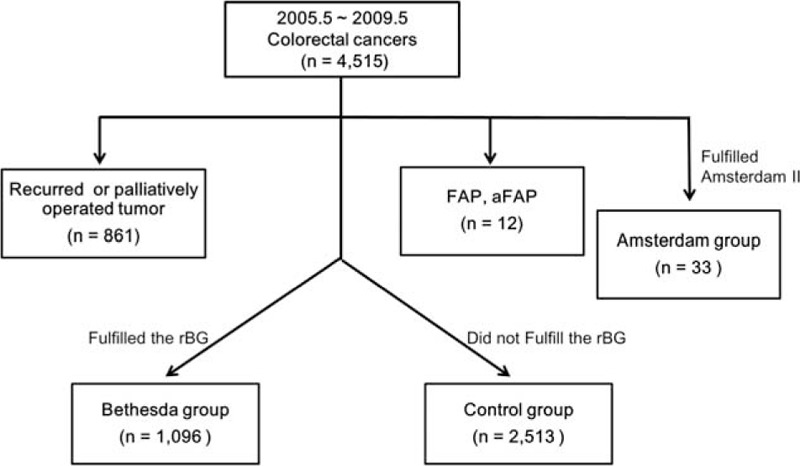
Overall study design and overview of patient population. aFAP = attenuated familial adenomatous polyposis coli, FAP = familial adenomatous polyposis coli, rBG = revised Bethesda guidelines.

Between May 2005 and May 2009, the medical records and databases of 4515 patients receiving surgery for CRC at the single institution were retrospectively reviewed. Primary CRCs pathologically confirmed as adenocarcinoma were identified in 3609 patients, and of these, 1096 (30.4%) patients were classified into the Bethesda group and 2513 (69.6%) patients were classified into the control group. Patients were excluded if they fulfilled the Amsterdam II criteria (Amsterdam group; n = 33), had familial adenomatous polyposis coli (FAP) or attenuated FAP (AFAP; n = 12 for both), or underwent palliative operation or reoperation for recurrent tumors (n = 861; Figure 1). Patients with rectal cancer who were treated with preoperative radiotherapy were excluded due to possible alteration in tumor DNA. The study was approved by the Asan Medical Center Institutional Review Board (IRB approval number: 2015-0655). Family history was obtained via a questionnaire and physician-led interview. Whenever possible, both maternal and paternal relatives were interviewed. The questionnaire included the family history of cancer in first-degree and second-degree relatives with regard to their current age, type of cancer, age at the time of diagnosis, diagnosing hospital, and current status. Nearly all hospitalized patients were interviewed by physicians. When some patients could not answer questionnaire and interview, their family members did. Relatives faintly recalled cancer types that were sometimes confirmed by the medical records from a related hospital. All patients were registered prospectively in the colorectal database and received close follow-up.

### Surgery

Radical curative surgery for CRC was performed. Curative surgery was defined as complete resection of any measurable disease with no involvement of the proximal, distal, and circumferential resection margin(s). All operations were performed by experienced colorectal surgeons (>500 colorectal surgeries).

### Assessment of Microsatellite Assay and Histological Examination

After surgery, a pathological examination was performed by specialized gastrointestinal pathologists. Staging was performed according to the American Joint Committee on Cancer (AJCC) 7th TNM classification of malignant tumors.^18^ In addition, differentiation, lymphovascular invasion (LVI), and perineural invasion (PNI) were documented. LVI and PNI were defined by current practice guidelines.^19–21^

Genomic DNA was extracted from microdissected non-neoplastic colon and representative tumor areas from 5 μm thick sections of formalin-fixed and paraffin-embedded tissues. MSI was determined by polymerase chain reaction (PCR), using primers amplifying the microsatellite markers BAT25 and BAT26 for mononucleotide repeats and D5S346, D2S123, and D17S250 for di-nucleotide repeats.^11^ Those showing instability in at least 2 of the markers were classed as MSI-H. Cases with no evaluable markers showing instability were classed as microsatellite stable (MSS), and the remainder was classed as MSI with low frequency (MSI-L). In the present study, 2823 (78.2%) cases of CRC were analyzed for MSI. IHC staining for hMLH1 and hMSH2 was performed in cases of CRC using diluted monoclonal antibodies against hMLH1 (G168-15; BD Pharmingen, San Diego, CA) and hMSH2 (G219-1129, BD Pharmingen). Normal colonic epithelium and lymphocytes, which exhibit strong nuclear staining for hMLH1 and hMSH2, were used as positive controls. In the present study, 3354 (92.9%) cases were analyzed for IHC staining for hMLH1 and hMSH2. The percentage of MMR-positive cells in a sample was divided into 2 grades according to nuclear staining: ≤10%, negative expression; >10%, positive expression. A d-MMR was defined as MSI-H and/or reduced expression of the MMR protein (either MLH1 and/or MSH2).

### Follow Up and Surveillance

The median follow-up was 82.9 (interquartile range, 72–101) months. Follow-up investigations included clinical examination, routine blood chemistry, serum carcinoembryonic antigen (CEA) screening, annual colonoscopy, chest radiography, and abdomino-pelvic and chest computed tomography (CT). We performed clinical examination with routine blood chemistry and CEA screening every 6 months for 2 to 3 years after operation and then annually thereafter. The first colonoscopy was postoperatively performed 6 months following surgery and repeated colonoscopy was performed at least every 2 years if no abnormal mucosal lesions were found. In the case of an abnormal lesion on colonoscopy, endoscopic biopsy is performed and the follow-up interval was shortened to 6 or 12 months. Abdomino-pelvic CT and chest CT were performed every 6 and 12 months, respectively, to identify relapse. Histopathological verification was performed where feasible. If metachronous CRC or HNPCC-related tumor occurred in patients during follow-up periods, the patients were censored as their recurrence free state.

### Statistical Analysis

Categorical variables were compared using the chi-squared or Fisher exact test, and continuous variables were compared using independent sample *t* tests. Logistic regression analysis was performed to calculate odds ratio (OR) and 95% confidence intervals (CIs) for demographic and clinicopathologic variables and of items of the revised Bethesda guidelines. Overall survival (OS) rates and recurrence-free survival (RFS) rates were expressed as percentages and analyzed using the Kaplan–Meier method. Survival curves were compared using the log-rank test. All statistical tests were 2-sided. The *P* values < 0.05 were considered statistically significant. Statistical analyses were performed using IBM SPSS Statistics for Windows, Version 21.0.0.0 (IBM Corp., Armonk, NY).

## RESULTS

### Clinicopathological Characteristics of Patients Fulfilling the Revised Bethesda Guidelines

Of the 1096 patients included in the Bethesda group, 170 fulfilled more than 2 items of the revised Bethesda guidelines (Table 1). Compared with the control group (n = 2513), the Bethesda group was younger (*P* < 0.001), had a higher frequency of right colon cancer, synchronicity and metachronicity (all *P* < 0.001), a lower frequency of synchronous adenoma (*P* < 0.001), and higher rate of reduced expression of MMR protein (hMLH1 and hMSH2; both *P* *<* 0.001) and MSI-H (*P* < 0.001) (Table 2). They also showed a more advanced T stage (*P* *=* 0.01), and poor differentiation (*P* < 0.01). Multivariate logistic regression analysis of variables between the revised Bethesda group and the control group revealed that younger age (OR = 0.76; 95% CI = 0.74–0.78; *P* *<* 0.001) and a higher rate of MSI-H (OR = 16.34; 95% CI = 9.07–29.43; *P* *<* 0.001) were independently discriminating factors (Table 3). In comparison with the Amsterdam group, the Bethesda group had a significantly lower frequency of right colon cancer (*P* *<* 0.01) and a lower rate of reduced expression of MMR protein (hMLH1, *P* *<* 0.01; hMSH2, *P* *<* 0.01) and MSI-H (*P* < 0.001) (see Table, Supplemental Digital Content 1, which demonstrates the comparison of clinicopathological parameters in the Bethesda group and the Amsterdam group). However, there was no significant difference between patients fulfilling more than 2 items of the revised Bethesda guidelines and patients in the Amsterdam group (see Table, Supplemental Digital Content 2, which demonstrates the comparison of clinicopathological parameters in patients fulfilling more than 2 items of revised Bethesda guidelines compared with the Amsterdam group).

**TABLE 2:**
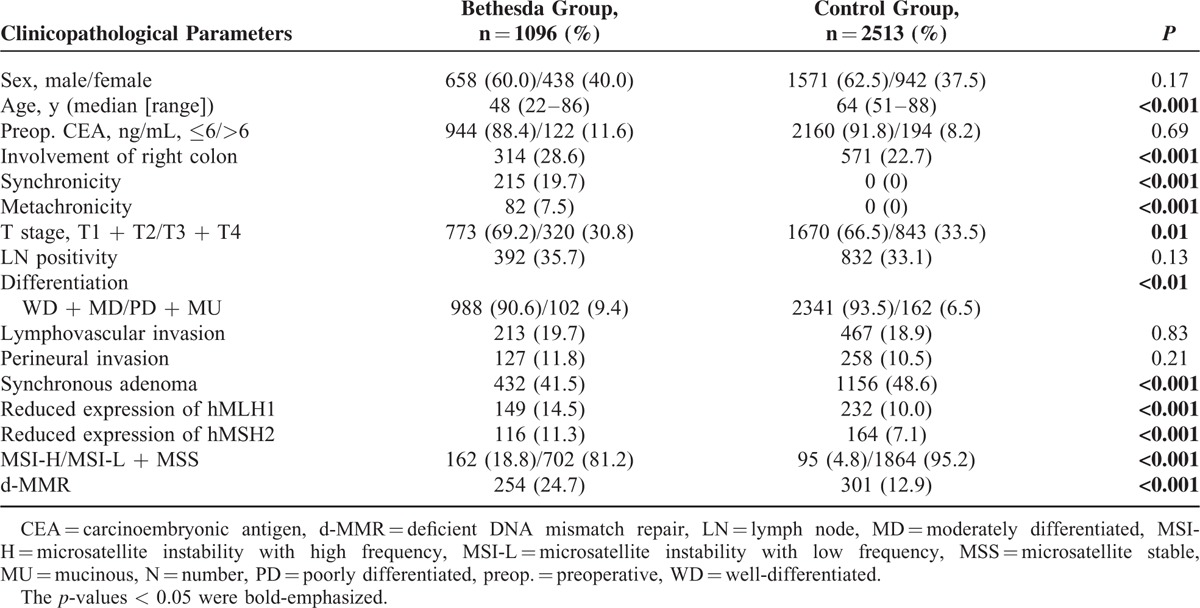
Comparison of Clinicopathological Parameters According to Fulfillment of the Revised Bethesda Guidelines

**TABLE 3:**
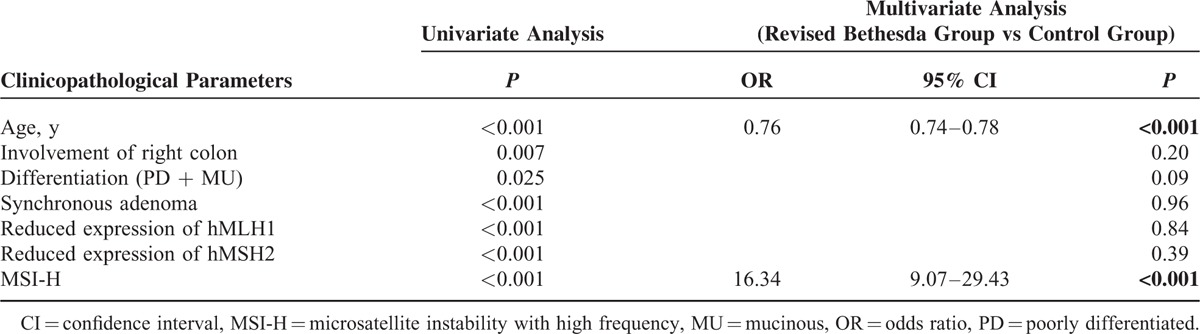
Univariate and Multivariate Analyses of Variables of the Bethesda Group Compared With the Control Group

Of the 1096 patients in the Bethesda group, 215 (19.7%) had synchronous CRC and/or HNPCC-related tumors, 82 (7.5%) had metachronous tumors, and 196 (17.9%) had synchronous and/or metachronous CRCs. Other detailed findings are summarized in Supplemental Digital Content 3 (see Table, which demonstrates the synchronicity and metachronicity in the Bethesda group).

### Performance Characteristics of the Revised Bethesda Guidelines

As a predictive marker of d-MMR tumors, the revised Bethesda guidelines showed a sensitivity of 63.0%, a specificity of 72.6%, a negative predictive value of 95.2%, and an overall accuracy of 71.8%. Furthermore, fulfillment of at least 2 items of the revised Bethesda guidelines demonstrated a sensitivity of 40.9% and a specificity of 98.3%. Lowered sensitivity was logically identified with an additional item fulfilling the revised Bethesda guidelines, while the specificity increased. Each individual item of the revised Bethesda guidelines had a high specificity (81.2–100.0%). In the Bethesda group, 951 (86.8%) patients who fulfilled item 1 and/or item 2 of the revised Bethesda guidelines showed a sensitivity of 42.8% and a specificity of 75.4% (Table 4).

**TABLE 4:**
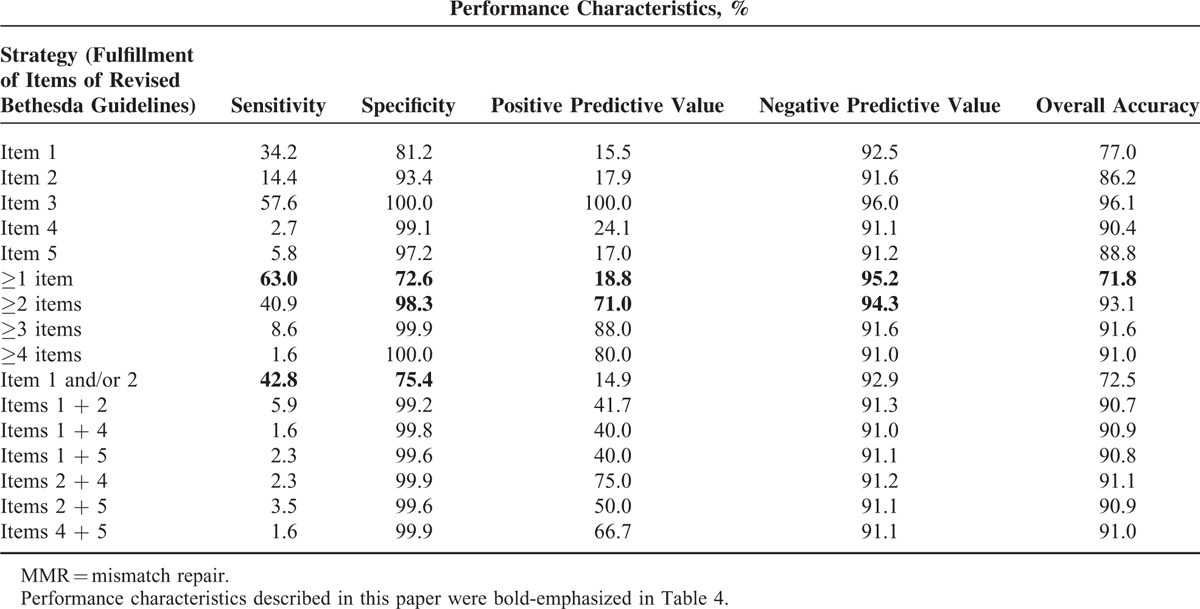
Performance Characteristics of Revised Bethesda Guidelines for the Identification of MMR Deficit of Tumor

Multivariate logistic regression analysis of individual items in the revised Bethesda guidelines as predictors of d-MMR (with the exception of item 3 including d-MMR such as MSI) revealed that items 1 and 2 were independently significant predictors of d-MMR (relative risk [RR] = 2.29, 95% CI = 1.49–2.43; RR = 2.23, 95% CI = 1.82–3.57; respectively; *P* *<* 0.001). Following the combination of individual items of the revised Bethesda guidelines to remove interacting confounding factors, fulfillment of items 1 and 2 (*P* *<* 0.001) and items 2 and 4 (*P* *=* 0.03) was an independently significant predictor of d-MMR (Table 5).

**TABLE 5:**
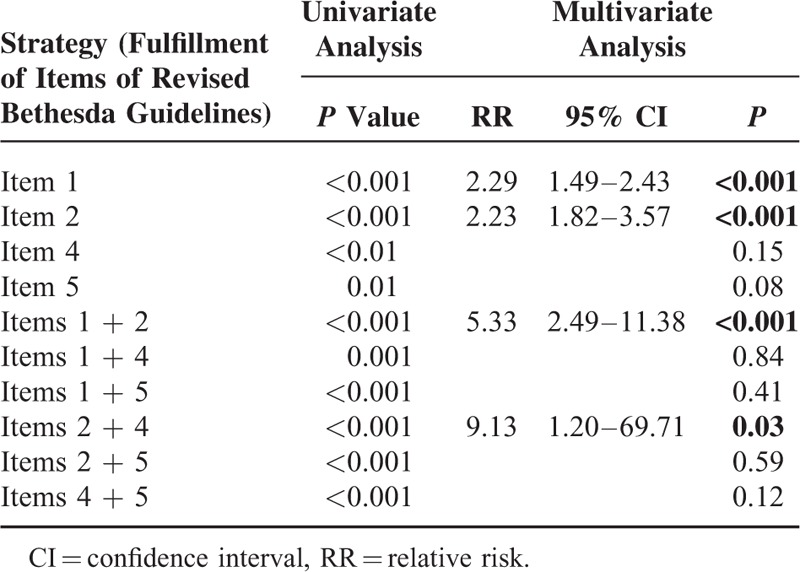
Univariate and Multivariate Analyses of Individual Items for the Demonstration of Deficient DNA Mismatch Repair With the Exception of Item 3

### Survival and Recurrence Period

**FIGURE 2:**
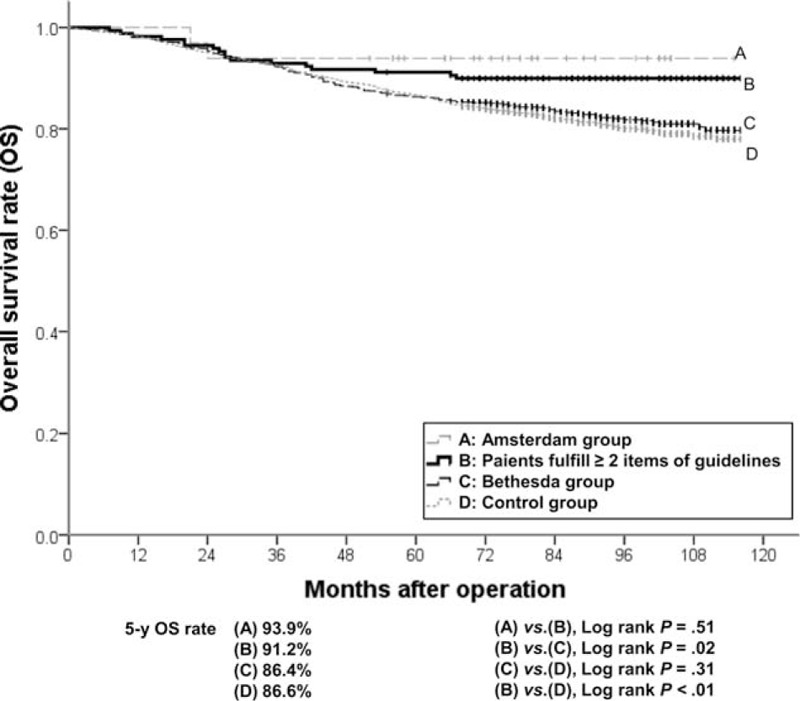
Cumulative overall survival rates of the (A) Amsterdam group, (B) Bethesda group fulfilling more than 2 items of the revised guidelines, (C) Bethesda group, and (D) control group.

**FIGURE 3:**
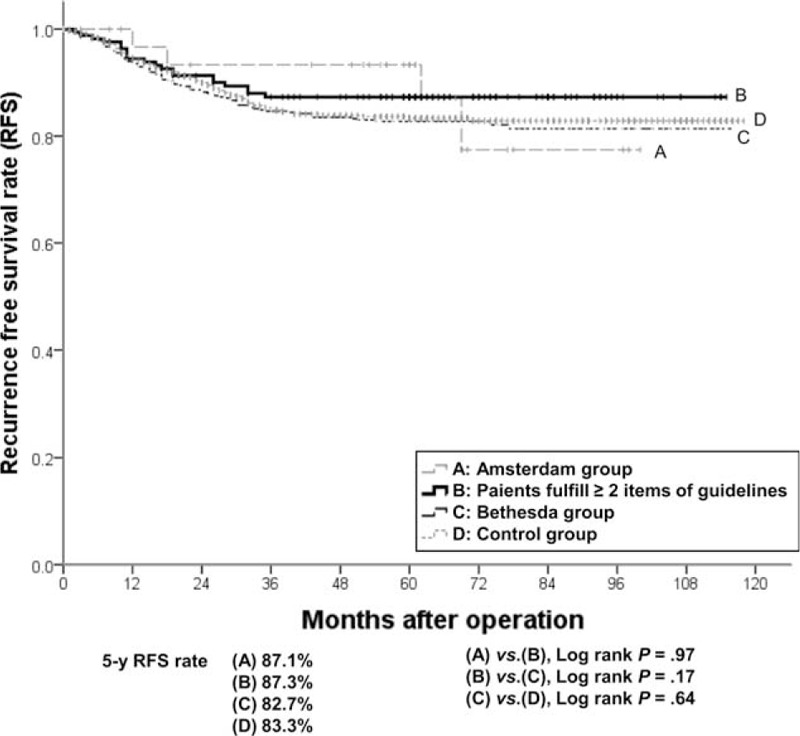
Recurrence-free survival rate of the (A) Amsterdam group, (B) Bethesda group fulfilling more than 2 items of the revised guidelines, (C) Bethesda group, and (D) control group.

The cumulative OS rates of the Bethesda group were not statistically different from those of the control group (5-year OS, 86.4% vs 86.6%, *P* = 0.31). Patients who fulfilled more than 2 items of the revised Bethesda guidelines showed significantly higher survival rates than the Bethesda group (5-year OS, 91.2% vs 86.4%, *P* = 0.02), and similar survival rates to the Amsterdam group (5-year OS, 91.2% vs 93.9%, *P* *=* 0.51; Figure 2). There was no significant difference in RFS rates between any of the 2 groups including the Bethesda group, the patients fulfilling more than 2 items of the revised Bethesda guidelines, and the control group (5-year RFS in the Bethesda group, the patients fulfilling ≥2 items of the revised Bethesda guidelines, and control groups: 82.7%, 87.3%, and 83.3%, respectively; Figure 3).

## DISCUSSION

We found that the Bethesda group showed young age, high rate of right colon cancer, MMR protein under-expression, MSI-H, and poorly differentiated. However, the Amsterdam group showed a greater association with these feature, compared with those in the Bethesda group. Fulfillment of more than 2 items of the revised Bethesda guidelines resulted in intensified clinicopathological features and higher survival rates, similarly with those in the Amsterdam group. We found that as a predictor of d-MMR, the revised Bethesda guidelines showed sound sensitivity and specificity in 3609 CRC patients, and that all individual items identified d-MMR with statistical significance. Taken together, the revised Bethesda guideline expressed clinicopathological characteristics of d-MMR CRCs^15,22^ and fulfillment of more than 2 items of the revised Bethesda guidelines strengthened the similarity with HNPCC.

Identification of patients most likely to carry a germline mutation in the *hMSH2*/*hMLH1* gene is important for diagnosis of HNPCC; however, universal molecular testing of all CRCs would be costly and time-consuming. The revised Bethesda guidelines only require a precise past medical and family history, with no additional cost. The present study reveals the fulfillment of more than any 2 items of the revised Bethesda guidelines resulted in a significantly high level of specificity for d-MMR in the absence of other pathological characteristics. However, its clinical applicability is low because there are excessive items in the revised Bethesda guidelines that require examination based on outpatients, a questionnaire, and an interview, particularly because items 4 and 5 which include history of relatives with HNPCC-related tumors in addition to CRC and younger age (<50 years) at diagnosis (Table 1) leads to a lack of precision. Furthermore, a recent trend of offspring reduction interferes with sufficient collection of corresponding relatives. Therefore, the revised Bethesda guidelines probably need to be more up to date and simple than the original guidelines. In the revised Bethesda guidelines, items 1 and 2 were extremely significant predictors of d-MMR, and multivariate analysis identified items 1 and 2 together as a significant pair among all 6 pairs of items examined. Furthermore, the majority of patients in the Bethesda group fulfilled item 1 and/or 2. The specificity for item 1 was slightly lower (81.2%) than that for the other items because all types of CRC, including the sporadic type, occurred at a younger age recently.^23^ In the present study, 7.1% of patients did not check for IHC staining for hMLH1 and hMSH2, 21.8% of patients did not check for MSI. IHC staining was primarily recommended in the first half of patients. Otherwise, some patients were not able to clarify genetic testing because samples were insufficient due to endoscopic mucosal resection of tumor.

According to the present study, the performance of the revised Bethesda guidelines was solid in terms of familial significance, and individual items had sufficient value for predicting d-MMR. In addition, the very highly negative predictive value of revised Bethesda guidelines presents the value of exclusion criteria for d-MMR, no further testing may be needed in patients who do not fulfill the revised Bethesda guidelines. MSI testing and/or IHC of MMR proteins must be performed to determine whether patients fulfill the revised Bethesda guidelines. A prospective study revealed that the fulfillment of revised Bethesda guidelines plus d-MMR showed sensitivity of 81.8%, specificity of 97.8% for MMR germline mutation.^15^ A combination of clinical guidelines (such as the revised Bethesda guidelines) and molecular biological testing (such as d-MMR) permits efficient surveillance and treatment. As analysis of all individual items is difficult, complex, and inaccurate, examination of items 1 and 2 alone may be efficient. Concerning operation, total colectomy might be initially recommendable as 196 (17.9%) patients incurred synchronous and/or metachronous CRC during follow-up. The high rate of synchronous and/or metachronous gastric cancer appears to be reflected by the high incidence of gastric cancer and some familial clustering of gastric cancer in Korea.^24^

This study recommends that Bethesda group with d-MMR undergo more frequent inspection of HNPCC-related organs, including the colon, for a period of more than 10 years according to their individual risk of HNPCC (in contrast to patients with sporadic CRC). Special physicians play a key role in identifying patients at high risk of HNPCC, and the ability to identify patients with a suspected cancer predisposition syndrome is crucial for rapid diagnosis and to ensure appropriate care.^25^

One potential weakness of this study is that the d-MMR of tumors is a bridge test and not a germline mutation test. The *MSH2*/*MLH1* germline mutation test was not formally evaluated during the study period. Otherwise, mean follow-up period was somewhat shorter (7 years), as more than 10 years are required to determine a precise oncological outcome. In addition, a prospective study in various cohorts must be needed to consolidate the item 1 and/or 2 representing the revised Bethesda guidelines.

## CONCLUSIONS

The clinicopathological features of patients fulfilling the revised Bethesda guidelines include young age, high rate of right colon cancer involvement, poor differentiation, MMR protein under-expression (hMLH1 and hMSH2), and MSI-H. The clinicopathological features of patients who fulfilled of more than 2 items of revised Bethesda guidelines appeared similar with those of patients who fulfilled Amsterdam II criteria. As a predictor of d-MMR, the revised Bethesda guidelines showed a sensitivity of 63.0%, a specificity of 72.6%, and a negative predictive value of 95.2%. Therefore, revised Bethesda guidelines showed the efficiency on predicting and exclusion for d-MMR. Items 1 and 2 were significant predictors of d-MMR and items 1 and 2 together were a significant pair among all 6 pairs examined. Examination of items 1 and 2 alone may be sufficient for successful application of revised Bethesda guidelines.

## Supplementary Material

Supplemental Digital Content
