# Supplementary material for: Observational Study: Familial Relevance and Oncological Significance of Revised Bethesda Guidelines in Colorectal Patients That Have Undergone Curative Resection
Source: Medicine (Baltimore). 2016 Feb 12;95(6):e2723. doi: 10.1097/MD.0000000000002723 (PMC4753907; doi:10.1097/MD.0000000000002723)
Supplement: Supplemental Digital Content [file medi-95-e2723-s001.docx]

**SUPPLEMENTAL DIGITAL CONTENT 1.** The Comparison of Clinicopathologic Parameters in the Bethesda Group and the Amsterdam Group.

| **Clinicopathological parameters** | **Bethesda group**  **n = 1,096 (%)** | **Amsterdam group**  **n = 33 (%)** | ***P*** |
| --- | --- | --- | --- |
| Sex, male / female | 658 (60.0) / 438 (40.0) | 13 (39.4) / 20 (60.6) | **.02** |
| Age, years [median (range)] | 48 ( 22–86 ) | 48 (22–75) | .21 |
| Involvement of right colon | 314 (28.6) | 17 (51.5) | **.01** |
| Synchronicity | 215 (19.7) | 7 (21.2) | .83 |
| Metachronicity | 81 (7.5) | 3 (6.1) | >.99 |
| T stage, T1 + T2/T3 + T4 | 773 (69.2) / 320 (30.8) | 8 (24.2) / 25 (75.8) | .70 |
| LN positivity | 392 (35.7) | 8 (24.2) | .20 |
| Differentiation |  |  | .51 |
| WD + MD / PD + MU | 988 (90.6) /102 (9.4) | 28 (96.6) / 1 (3.4) |  |
| Lymphovascular invasion | 213 (19.7) | 3 (9.1) | .35 |
| Perineural invasion | 127 (11.8) | 3 (9.1) | .19 |
| Synchronous adenoma | 432 (41.5) | 16 (50.0) | .37 |
| Reduced expression of hMLH1 | 149 (14.5) | 11 (35.5) | **< .01** |
| Reduced expression of hMSH2 | 116 (11.3) | 9 (29.0) | **< .01** |
| MSI-H /MSI-L + MSS | 162 (18.8) / 702 (81.2) | 19 (67.9) / 9 (32.1) | **< .001** |
| d-MMR | 254 (24.7) | 22 (71.0) | **< .001** |

N = number, LN = lymph node, WD = well-differentiated, MD = moderately differentiated, PD = poorly differentiated, MU = mucinous, MSI-H = microsatellite instability with high frequency, MSI-L = microsatellite instability with low frequency, MSS = microsatellite stable, d-MMR = deficient DNA mismatch repair.

**SUPPLEMENTAL DIGITAL CONTENT 2.** The Comparison of Clinicopathologic Parameters in Patients Fulfilling More Than 2 Items of Revised Bethesda Guidelines Compared with the Amsterdam Group.

| **Clinicopathological parameters** | **Patients fulfilling ≥ 2 items of the RBG**  **n = 170 (%)** | **Amsterdam group**  **n = 33 (%)** | ***P*** |
| --- | --- | --- | --- |
| Sex, male / female | 104 (61.2) / 66 (38.8) | 13 (39.4) / 20 (60.6) | **.04** |
| Age, years [median (range)] | 46 (26–81) | 48 (22–75) | .37 |
| Involvement of right colon | 86 (50.6 ) | 17 (51.5) | .69 |
| Synchronicity | 53 (31.5) | 7 (21.2) | .30 |
| Metachronicity | 27 (16.1) | 2 (6.1) | .64 |
| T stage, T1 + T2/T3 + T4 | 34 (30.2) / 134 (79.8) | 8 (24.2) / 25 (75.8) | .64 |
| LN positivity | 51 (30.0) | 8 (24.2) | .68 |
| Differentiation |  |  | .14 |
| WD + MD / PD + MU | 144 (84.7) / 26 (15.3) | 28 (96.6) / 1 (3.4) |  |
| Lymphovascular invasion | 28 (16.6) | 3 (9.1) | .50 |
| Perineural invasion | 11 (6.5) | 3 (9.1) | .72 |
| Synchronous adenoma | 60 (36.6) | 16 (50.0) | .17 |
| Reduced expression of hMLH1 | 60 (36.6) | 11 (35.5) | > .99 |
| Reduced expression of hMSH2 | 48 (29.3) | 9 (29.0) | > .99 |
| MSI-H /MSI-L + MSS | 105 (70.9) / 43 (29.1) | 19 (67.9) / 9 (32.1) | .82 |
| d-MMR | 120 (73.2) | 22 (71.0) | .83 |

RBG = revised Bethesda guidelines; N = number, LN = lymph node, WD = well-differentiated, MD = moderately differentiated, PD = poorly differentiated, MU = mucinous, MSI-H = microsatellite instability with high frequency, MSI-L = microsatellite instability with low frequency, MSS = microsatellite stable, d-MMR = deficient DNA mismatch repair.

**SUPPLEMENTAL DIGITAL CONTENT 3.** The Synchronicity and Metachronicity in the Bethesda Group (n = 1,096).

| ***HNPCC-related tumor** | **Synchronous tumor**  **n = 215 (%)** | **Metachronous tumor**  **n = 82 (%)** | **Syn. and/or Met. tumor n = 281 (%)** |
| --- | --- | --- | --- |
| Colorectal cancer | 164 (76.3) | 40 (48.8) | 196 (69.8) |
| Stomach cancer | 55 (25.6) | 32 (39.0) |  |
| Endometrial cancer | 4 (1.9) | 4 (4.9) |  |
| Ovary cancer | 3 (1.4) | 1 (1.2) |  |
| Renal cell carcinoma | 11 (5.2) | 4 (4.9) |  |
| Ureter cancer | 4 (1.9) | 4 (4.9) |  |
| Bladder cancer | 3 (1.4) | 4 (4.9) |  |
| Malignancy of biliary system | 10 (4.7) | 1 (1.2) |  |

HNPCC = hereditary non-polyposis colorectal cancer, n = number, syn.= synchronous HNPCC-related tumor, met. = metachronous HNPCC-related tumor, *HNPCC-related tumors include colorectal cancer.
